# Field-Template, QSAR, Ensemble Molecular Docking, and 3D-RISM Solvation Studies Expose Potential of FDA-Approved Marine Drugs as SARS-CoVID-2 Main Protease Inhibitors

**DOI:** 10.3390/molecules26040936

**Published:** 2021-02-10

**Authors:** Poonam Kalhotra, Veera C. S. R. Chittepu, Guillermo Osorio-Revilla, Tzayhri Gallardo-Velazquez

**Affiliations:** 1 Departamento de Biofísica, Escuela Nacional de Ciencias Biológicas, Instituto Politécnico Nacional, Prolongación de Carpio y Plan de Ayala S/N, Col. Santo Tomás, Ciudad de México 11340, CP, Mexico; kalhotrapoonam@gmail.com or; 2 Departamento de Ingeniería Bioquímica, Escuela Nacional de Ciencias Biológicas, Instituto Politecnico Nacional, Av. Wilfrido Massieu S/N, Col. Unidad Profesional Adolfo López Mateos, Zacatenco, Ciudad de México 07738, CP, Mexico; veerareddy9@gmail.com or

**Keywords:** SARS-CoV-2, CoVID-2 main protease, FDA-approved marine drugs, ensemble molecular docking, field-template models, antivirals

## Abstract

Currently, SARS-CoV-2 (severe acute respiratory syndrome coronavirus 2) has infected people among all countries and is a pandemic as declared by the World Health Organization (WHO). SARS-CoVID-2 main protease is one of the therapeutic drug targets that has been shown to reduce virus replication, and its high-resolution 3D structures in complex with inhibitors have been solved. Previously, we had demonstrated the potential of natural compounds such as serine protease inhibitors eventually leading us to hypothesize that FDA-approved marine drugs have the potential to inhibit the biological activity of SARS-CoV-2 main protease. Initially, field-template and structure–activity atlas models were constructed to understand and explain the molecular features responsible for SARS-CoVID-2 main protease inhibitors, which revealed that Eribulin Mesylate, Plitidepsin, and Trabectedin possess similar characteristics related to SARS-CoVID-2 main protease inhibitors. Later, protein–ligand interactions are studied using ensemble molecular-docking simulations that revealed that marine drugs bind at the active site of the main protease. The three-dimensional reference interaction site model (3D-RISM) studies show that marine drugs displace water molecules at the active site, and interactions observed are favorable. These computational studies eventually paved an interest in further in vitro studies. Finally, these findings are new and indeed provide insights into the role of FDA-approved marine drugs, which are already in clinical use for cancer treatment as a potential alternative to prevent and treat infected people with SARS-CoV-2.

## 1. Introduction

The World Health Organization (WHO) has declared ongoing outbreak severe acute respiratory syndrome coronavirus 2 (SARS-CoV-2) as a pandemic, where SARS-CoV-2 is closely related to earlier coronaviruses SARS-CoV, and this new virus has infected millions of people [1]. Phylogenetic analysis of the SARS-CoV-2 virus reveals its high similarity or resemblance to previous coronaviruses such as SARS-CoV-1 and MERS-CoV [2]. The new coronavirus first detected in China infected more than 102,303,716 people worldwide and with more than 2,212,694 deaths worldwide by January 30, 2021 (according to webpage coronavirus.jhu.edu). It is also observed that the mortality rate of SARS-CoV-2 virus-infected patients is lower, but its transmission rate is higher, than previously reported coronaviruses [3]. SARS-CoV-2 genome analysis revealed several structural proteins that are targeted using computationally aided drug-discovery methods to develop antiviral drug candidates. Among therapeutic drug targets, the well-characterized structural protein in a complex with inhibitors using the X-ray crystallography technique is the CoVID-19 main protease [4,5].

CoVID-19 main protease also referred to as 3CL protease (M pro), digests viral polyproteins pp1 a and pp1 b (L-Q*(S, A, G) (* shows the cleavage site)). These polyproteins are essential for the transcription and translation of coronavirus [6], thus the inhibition of the functional activity of CoVID-19 main protease offers an excellent strategy for critical defense and antiviral drug production. The CoVID-19 main protease has a specific substrate for glutamine, which is distinct to host proteases and provides an opportunity for developing targeted therapeutics (antivirals) by targeting the CoVID-19 main protease [7].

The active site is located on the main CoVID-19 main protease, the protease forms a homodimer, and the binding pocket lies in two domains: domain 1 and domain 2 [8]. In addition, many crystal structures were determined for CoVID-19 main protease in complex with a different class of inhibitors, that eventually had given a clue of critical regions and interacting residues involved in CoVID-19 leading-protease catalytic site and site role in inhibition.

The structure–activity relationship (SAR) on protease inhibitors provides the insights into different binding conformations and an opportunity to apply computationally aided drug-discovery and drug-development methods (CADD) [9,10]. CADD methods had reduced the timeline and the costs to develop the drug and to reach clinical use significantly compared to traditional drug-discovery and drug-development methods [11,12,13]. Despite advances in CADD technology, challenges like time for a drug to reach clinical use and related costs are some problems that must be overcome on timescale. These problems lead academia and industries to offer technology that repurposes FDA-approved drugs for new treatments [14].

Structural, diversity, and complexity molecular features are seen in drugs derived from marine origin and are worth looking into for the potential in the discovery of chemicals/drugs for CoVID-19 main-protease inhibitors [15]. Hence, in the current study, CADD methods were used to understand the potential of FDA-approved marine drugs as CoVID-19 main-protease inhibitors. So, initially ligand-based drug-discovery methods, such as the field-template [16] and activity-atlas model [17,18,19], were used to understand the structural features as a function of electrostatics, hydrophobics, and activity shapes responsible for CoVID-19 protease inhibition. The ensemble molecular-docking method was used to study the binding at the active site and interacting residues, where this method was used to understand the binding of approved marine drugs against the main-protease-binding site [20,21,22,23]. Finally, we conducted solvation effects on the binding of drugs using the three-dimensional reference interaction site model (3D-RISM) method, which eventually showed the FDA-approved marine drugs as promising results [24,25,26].

## 2. Results and Discussion

### 2.1. Pharmacophore Model (Field Template) of SARS-CoVID-19 Main-Protease Inhibitors

**Figure 1:**
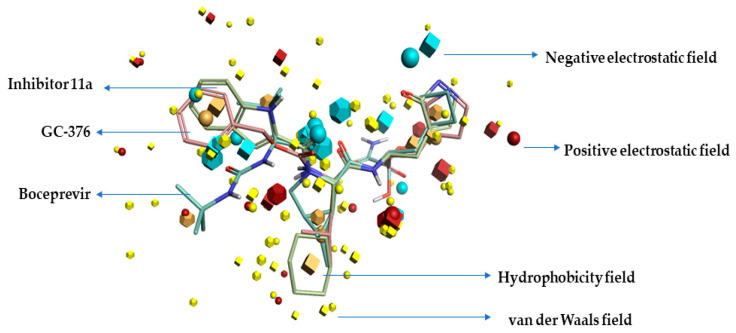
Field template of SARS-CoVID-19 main-protease inhibitors generated using field template tool.

In this study, three compounds named GC-376, Inhibitor-11a, and boceprevir proven to be SARS-CoVID-19 main-protease inhibitors were used, and structures have been retrieved to study the three-dimensional bioactive conformations and as well the described field template (also called the pharmacophore hypothesis). The top-scoring/ranking template showed 62.7 percent similarity (general), 55.1 percent field similarity, and 70.3 percent shape similarity, to which the SARS-CoVID-19 main-protease inhibitors were aligned, eventually revealing the structure–activity relationship model of SARS-CoV-2 main-protease inhibitors. Figure 1 displays the resultant field template of SARS-CoVID-19 main-protease inhibition features. All the generated fields have been visualized using the Forge visualization tool, and the template represents the consensus alignment, as well bioactive conformations of three chemicals.

### 2.2. Structure–Activity Relationship Model of SAR-CoVID-19 Main-Protease Inhibitors

To gain molecular insights into the FDA-approved marine drugs that possess CoVID-19 main-protease-inhibitor features and to further optimize their efficiency and potency, and to help in identifying new natural inhibitors of SARS=CoVID-19 main-protease enzyme, the structure–activity relationship (S.A.R.) was studied using the technology activity-atlas model. Initially, an average of actives, activity cliff summary, and potential regions of activity was studied for proven SARS-CoVID-19 main-protease inhibitors.

**Figure 2:**
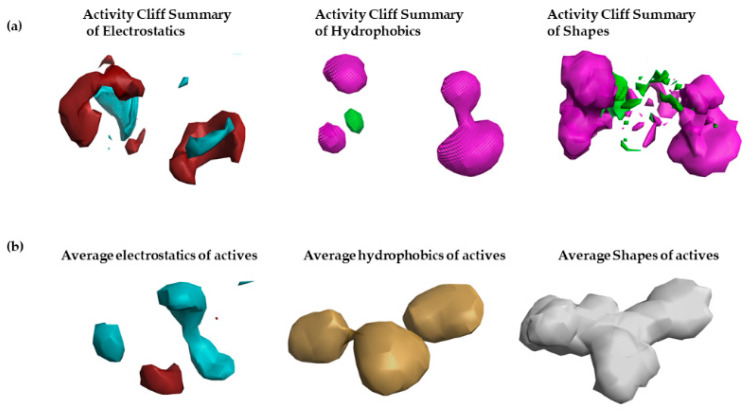
Alignment of 19 SARS-CoVID-19 main-protease inhibitors to field-template-generated activity-atlas model which provides structure-activity relationship: (**a**) Activity Cliff summary of electrostatics, hydrophobics, and shapes; (**b**) Average of actives. All features are visualized using the Forge visualization tool, which led to understanding the structure-activity relationship of different compounds proven to inhibit SARS-CoVID-19 main-protease inhibitors that are proven experimentally.

To construct the activity-atlas model (structure–activity relationship), 19 CoVID-19 main-protease inhibitors (shown in Table 1), with inhibitory IC50 (µM) values, were collected from the previous literature [5,8,27]. The structures are retrieved from PubChem, and all chemicals are aligned to previously generated pharmacophore or field-template model of CoVID-19 main-protease inhibitors. The resultant activity-atlas model is shown in Figure 2. The built activity-atlas model (S.A.R. model) provides detailed molecular insights about the activity cliff summary of positive (maroon) and negative (blue) electrostatics, a summary of favorable (green) and unfavorable hydrophobic (pink), and summary of favorable and unfavorable shapes (see Figure 2a). The S.A.R. model also provides information on average electrostatics, hydrophobics, and shapes of actives corresponding to CoVID-19 main protease inhibition.

The results revealed that electropositive field regions observed within the activity-atlas model are responsible for CoVID-19 main-protease inhibition. Increased or higher field regions reveal an increased association with inhibitory activity. Finally, the resultant model was used to screen three FDA-approved drugs, Eribulin Mesylate, Plitidepsin, and Trabectedin, which are derived from a marine source, and to assign a novelty score to them and calculate their potential to be CoVID-19 main-protease inhibitors.

### 2.3. Molecular Features of FDA-Approved Marine Drugs as CoVID-19 Main-Protease Inhibitors

**Figure 3:**
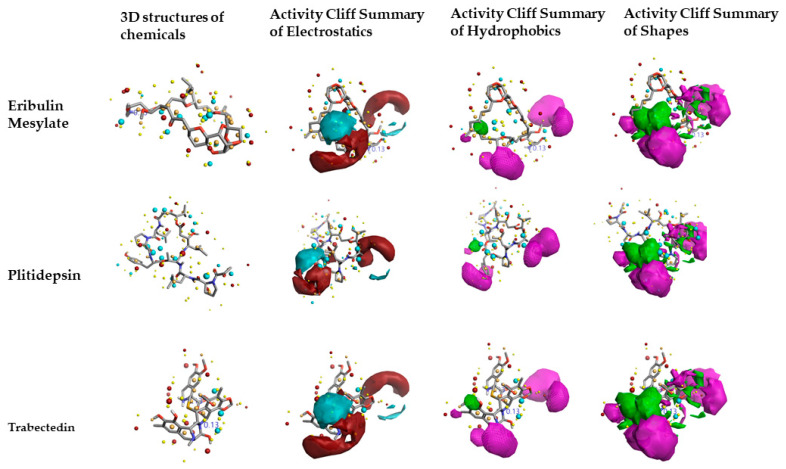
The electrostatics, hydrophobics, and activity-shape features of three FDA-approved drugs, Eribulin Mesylate, Plitidepsin, and Trabectedin, which are derived from a marine source predicted using activity-atlas model of CoVID-19 protease inhibitors. All features were visualized using the Forge visualization tool.

Figure 3 shows the results predicted by activity-atlas model on three FDA-approved drugs Eribulin mesylate, Plitidepsin, and Trabectedin inhibitory features similar to already proven ones that inhibit the CoVID-19 main-protease function. The Cresset XED force field was used to calculate important features of the chosen drugs. Positive and negative electrostatic, hydrophobic features of Plitidepsin are slightly different from Eribulin mesylate and Trabectedin, but all observed features belong to the regions of CoVID-19 main-protease inhibitors.

Activity-atlas model results revealed that Eribulin mesylate possessed a novelty score of “very high”, and other chemicals as “low”. The calculated score “very high” reveals the drug Eribulin mesylate contains pharmacophore features overly different from already-proven CoVID-19 main-protease inhibitors, and the low score means it is similar to the features already proven to be inhibitors. The minor changes observed in fields and scores can influence the binding affinity to the CoVID-19 main protease, and for this reason, they were studied using protein–ligand interactions using ensemble molecular-docking simulations.

### 2.4. Ensemble Molecular-Docking Simulations

In this study, the ensemble molecular-docking tool provided by Flare software was used. Initially, the docking protocol was validated using a redocking methodology. Later, X-ray crystal structures related to CoVID-19 main protease in complex with inhibitors were retrieved from the protein data bank web server, and the grid was constructed around the binding site of CoVID-19 main-protease inhibitors. Upon construction of the grid, three chemicals, Eribulin Mesylate, Plitidepsin, and Trabectedin, that were approved by the FDA were docked and the resulting complexes were scored using the Flare docking algorithm. The binding free-energy analysis among the three chemicals revealed their excellent binding affinity to SARS-CoVID-19 main protease. The basic interacting residues involved at the binding site were hydrogen bonding, van der Waals, alkyl, and Pi-alkyl.

**Figure 4:**
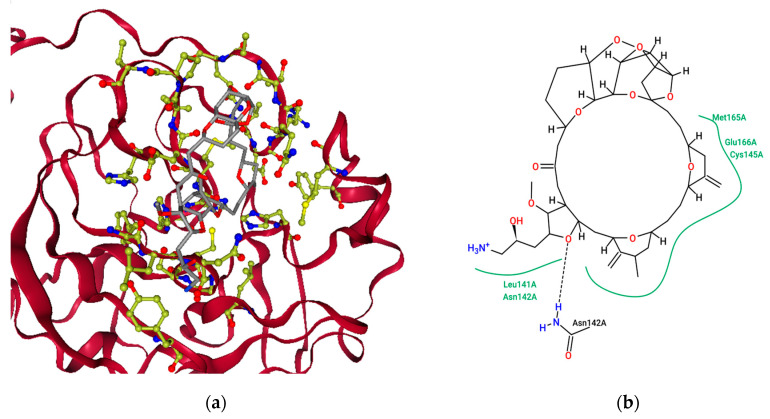
The binding pose view of Eribulin mesylate at the active site of the SARS CoVID-19 main protease (**a**), and (**b**) residues involved at the binding site.

Figure 4a represents the chemical Eribulin mesylate’s best-scoring binding pose in complex with SARS-CoVID-19 main protease with a docking score of −11.304 kcal mol^−1^, and Figure 4b shows the key interacting residues at the binding pose. To gain insights into the residues responsible for negative free energy, a pose view was used, and the key residues seen were HIS 41, MET 49, TYR 54, PHE 140, LEU 141, ASN 142, SER 144, CYS 145, HIS 163, HIS 164, MET 165, GLU 166, LEU 167, PRO 168, HIS 172, ASP 187, ARG 188, GLN 189, THR 190, ALA 191, and GLN 192. Further insights into non-bonded interactions revealed that the chemical Eribulin mesylate makes strong hydrogen-bonding interactions with interacting residues ASN 142 and GLN 189, alkyl interactions with MET 165, and pi-alkyl interactions HIS 41 and 163 with the active site of SARS-CoVID-19 main protease. The biological half-life time of Eribulin mesylate is 40 h [28].

**Figure 5:**
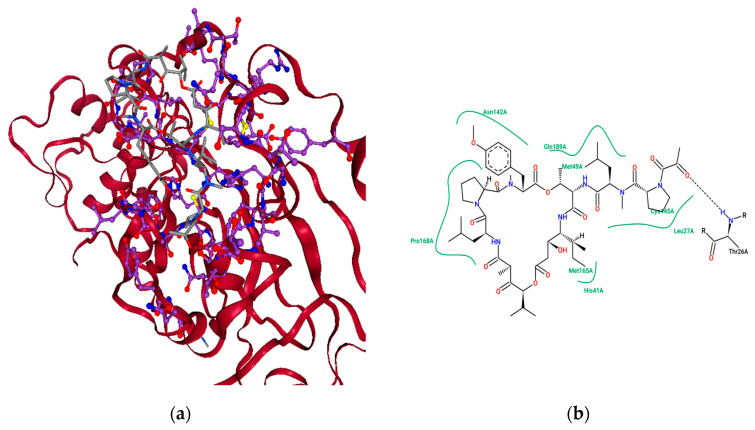
The binding pose view of Plitidepsin at the active site of the SARS CoVID-19 main protease (**a**), and (**b**) residues involved at the binding site.

Similarly, binding studies of the marine-drug Plitidepsin and CoVID-19 main protease were studied using the Flare docking algorithm, and Figure 5a reveals the best-scoring binding poses in complex with SARS-CoVID-19 main protease with a docking score of −13.572 kcal mol^−1^, and Figure 5b shows the key interacting residues at the binding pose. To gain insights into interacting residues responsible for negative free energy, the discovery studio visualizer was utilized, and the key residues seen were THR 25, THR 26, LEU 27, HIS 41, VAL 42, SER 46, MET 49, LEU 141, ASN 142, GLY 143, SER 144, CYS 145, HIS 163, HIS 164, MET 165, GLU 166, PRO 168, GLY 170, ARG 188, GLN 189, THR 190, and GLN 192. Further insights into nonbonded interactions revealed that the chemical Plitidepsin makes strong hydrogen-bonding interactions with residues THR 26, HIS 41, GLU 166, THR 190, THR 25, MET 165, GLN 189, ASN 142, and LEU 141; alkyl interactions with CYS 145, PRO 168, MET 165, LEU 27 and MET 49; pi-sulfur interactions with CYS 145; and pi-alkyl interactions with HIS 41 with the active site of SARS-CoVID-19 main protease. The biological half-life of Plitidepsin is 21–44 h [29]. Recently, the drug plitidepsin was proven to possess antiviral activity on SARS-CoV-2 virus in invitro with 27.5-fold times higher than Remdesivir, with less toxicity [30]. This study would reveal the new possible mechanism of action that could explain its therapeutic role to reduce the viral replication.

**Figure 6:**
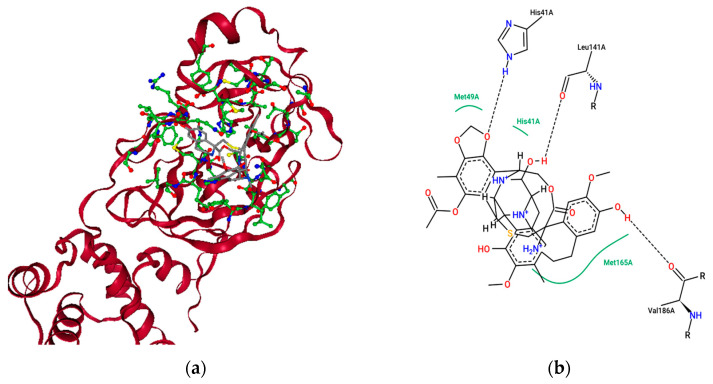
The binding pose view of Trabectedin at the active site of the SARS CoVID-19 main protease (**a**), and (**b**) residues involved at the binding site.

Figure 6a represents the chemical Trabectedin’s best-scoring binding poses in complex with SARS-CoVID-19 main protease with a docking score of −12.032 kcal mol^−1^, and Figure 6b shows the key interacting residues at the binding pose. To gain insights into residues responsible for negative free energy, the discovery studio visualizer was used, and the key residues seen were LEU 27, HIS 41, VAL 42, CYS 44, MET 49, LEU 141, GLY 143, CYS 145, HIS 163, HIS 164, PHE 181, VAL 186, ASP 187, THR 190, and GLN 192. Further insights into nonbonded interactions revealed that the chemical Trabectedin makes strong hydrogen-bonding interactions with residues HIS 41, ASN 142, LEU 141, VAL 186, and HIS 164; pi-sigma interactions with GLN 189; alkyl interactions with LEU 27, M.E.T. 49, and MET 165; and pi-alkyl interactions with HIS 41, HIS 164 and MET 49 with the active site of SARS-CoVID-19 main protease. The biological half-life of Trabectedin is 27–89 h [31].

### 2.5. Solvation Studies on FDA-Approved Marine Drugs Bound with CoVID-19 Main Protease

**Figure 7:**
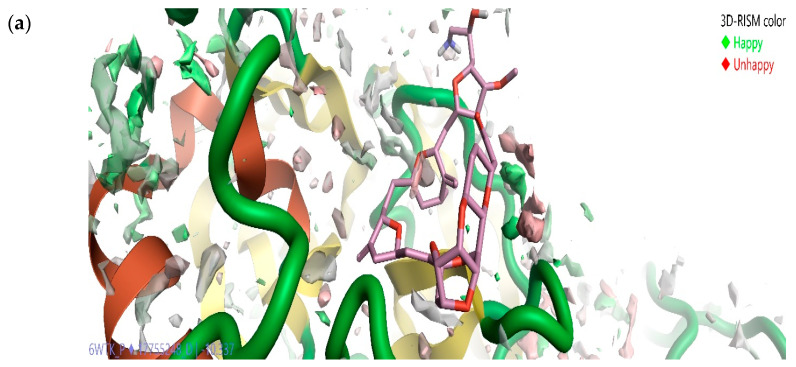

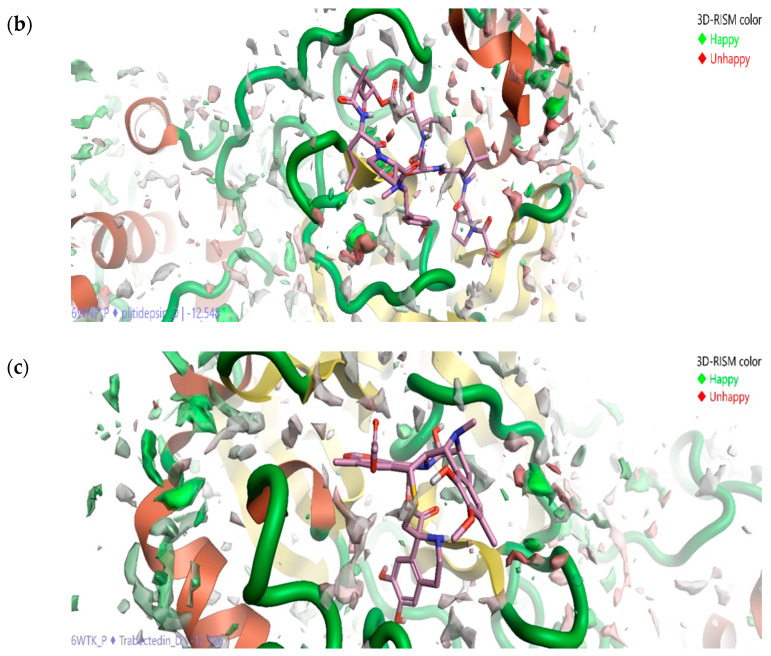
Solvation studies on Eribulin mesylate (**a**), Plitidepsin (**b**), and Trabectedin (**c**) in complex with SARS-CoVID-19 main protease using a three-dimensional reference interaction site model (3D-RISM), where results reveal oxygen isodensity surface at ρ = 5.

The stability of biomacromolecules in bulk water molecules generated by Flare molecular docking calculations at the active site was studied using a three-dimensional reference interaction site model (3D-RISM) algorithm. High water density was formed at the active site of SARS-CoVID-19 main protease at the end of 3D-RISM calculations. Figure 7a shows the oxygen density and hydrogen density of Eribulin mesylate bound at the active site of SARS-CoVID-19 main-protease enzyme with favorable (green spheres denote the molecule is happy), and unfavorable (red spheres denote the molecule is unhappy). The solvation studies reveal that drugs binding at the active site of CoVID-19 main protease are stable in the presence of bulk water. The calculations reveal that the green-color-labeled molecules (happy) are difficult to be displaced by the marine drug Eribulin mesylate, and the one with the red (unhappy) is easily displaced (Figure 7). This study helped gain molecular insights into the binding site and also helped in improving the precision of how Eribulin mesylate binds at the active site of SARS-CoVID-19 main protease. This information can also be used to perform Eribulin mesylate to enhance the potency of CoVID-19 main-protease inhibition.

Similarly, Figure 7b shows the oxygen density and hydrogen density of Plitidepsin bound at the active site of SARS-CoVID-19 main-protease enzyme with favorable (green spheres) and unfavorable (red spheres). The presence of spheres reveals that binding by the plitidepsin compound at the active site is stable in the presence of bulk water. The calculations reveal that the green-color-labeled molecules are difficult to be displaced by the marine drug Plitidepsin, and the one with the red color is easily displaced (Figure 7). The solvation study helped in gaining more molecular insights into the binding sites of CoVID-19 main protease and also helped in improving the precision of how the marine drug plitidepsin binds at the active site of SARS CoVID-19 main protease. This information can also be used to perform Plitidepsin derivatives, and eventually improve its potency in case of CoVID-19 main-protease inhibition. Figure 7c shows the oxygen density and hydrogen density of Trabectedin bound at the active site of SARS-CoVID-19 main-protease enzyme with favorable (green spheres) and unfavorable (red color spheres). This reveals that binding is stable in the presence of bulk water. The calculations reveal that the green-color-labeled molecules are difficult to be displaced by the marine drug Trabectedin, and the one with the red color is easily displaced (Figure 7c). This study helped in gaining molecular insights into the binding site and helped in improving the precision of how Trabectedin binds at the active site of SARS-CoVID-19 main protease. This information can also be used to perform Trabectedin and to improve the potency of CoVID-19 main-protease inhibition. All the drugs are proven to be stable in the presence of bulky water and possess favorable and unfavorable regions.

## 3. Materials and Methods

### 3.1. Activity Atlas Model of SARS-CoV-2 Main-Protease Inhibitors

Initially, the pharmacophore hypothesis was designed and studied using the Forge software (Cresset Inc., Cambridgeshire, UK). The generated theory was based on existing inhibitors (seven) of SARS-CoV-2 main-protease inhibitors. The generated pharmacophore hypothesis was constructed around the hydrophobic shape, sizes, van der Waals shapes, and electrostatic features (positive and negative) representing the overall functional activity as SARS-CoV-2 main-protease inhibitors. The generated pharmacophore hypothesis template was used to apply the Bayesian approach and generate the activity-atlas model of SARS-CoV-2 main-protease inhibitors. The generated activity-atlas model was then used to understand the potential of FDA-approved marine drugs that possess the standard features responsible for protease inhibition. At this point in time, twelve drugs derived from a marine source were approved by the FDA: namely, four nucleosides (Cytarabine, Fludarabine phosphate, Nelarabine, and Vidarabine), two antibiotics (Cephalosporin C and Rifamycin), one antibody drug conjugate (Brentuximab Vedotin), one cyclic peptide (Ziconotide), and three small molecules (Trabectedin, Eribulin Mesylate, and Plitidepsin). In this study, three small molecules were chosen to understand the potential and critical features of CoVID-19 main-protease inhibition, which were visualized using Forge visualization software.

### 3.2. Ensemble Molecular-Docking Simulations

The X-ray crystal structure of SARS-CoV-2 main protease complexed with inhibitors Carmofur (PDB ID: 7BUY), Boceprevir (PDB ID: 7BRP), N3 (PDB ID: 7BQY), and Feline (PDB ID: 6WTK) was utilized to perform molecular-docking simulations with the lead finder algorithm [32]. Initially, all the retrieved complexes are validated using the SAVES server (https://saves.mbi.ucla.edu/). Later, the protein-preparation module provided by the Flare tool was utilized to load the complexes and perform ensemble-docking calculations. The conditions used in this analysis for docking have been validated using redocking methodology [33]. In brief, the inhibitors were docked back to original receptor, and the lead finder algorithm could reproduce the binding pose, similar to the complex obtained using crystallography technique whose root mean square deviation (RMSD) is equal to 1.5 Å. Initially, the grid box was constructed around reference ligands to span 10 Å in each direction. For each marine drug, ten different poses were generated, and the lead finder algorithm determined the free energy of binding and the least binding energy. The top-ranking pose was chosen to further study interacting residues involved between the protease and FDA-approved marine drugs. Protein–ligand interactions were analyzed using the Protein Plus server [34].

### 3.3. Solvation Studies Using 3D R.I.S.M.

The Flare tool was utilized to perform the solvation studies on marine drugs bound to SARS-CoV-2 main protease, where a three-dimensional reference interaction site model (3D-RISM) approach was used. A 3D-RISM approach is a new approach to solvation studies that helps to analyze stability and the structure of water molecules in and around the binding of the drug at CoVID-19 main-protease active site based on the molecular Ornstein–Zernike equation [35]. In this study we used KH closure, and the Equation (1) reveals the total correlation function *h*(*r*_12_) among particles as contribution from a direct correlation (*r*_12_) and contributions from chains of mediating particles that are responsible for the total integral sum [35]. DFT calculations with ONETEP and interaction statistics from the CSD were used to calculate water positions and energetics around a range of a small molecule model. The use of XED force field led to utilize polarization and electronic anisotropy in assessing water structure around carbonyl groups, that is proven to have better accuracy on 3D-RISM calculation around the active site of CoVID-1 main protease [36].

(1) $$h\left(r_{12}\right)=c\left(r_{1.2}\right)+\int dr_{3}C\left(r_{13}\right)\rho \left(r_{3}\right)h\left(r_{23}\right)\;$$

Statistical mechanics was applied to solve Equation (1) by using a suitable closure relation, and it is iterated until self-consistency is achieved. To solve the above equation, the Flare tool was utilized. The tool provides the output as a grid which is composed of individual densities. One density for oxygen and another for hydrogen are provided for an individual water molecule. Free energy was assigned to each value and denoted as favorable (happy and denoted in green) and unfavorable (unhappy and denoted in red).

In this study, we utilized Cresset’s X.E.D. forcefields on marine drugs bound to CoVID-19 main-protease complexes, and the resulting isosurfaces calculations revealed molecular insights into solvation studies.

## 4. Conclusions

In this research article, computational techniques including field-template, QSAR, ensemble molecular docking, and 3D-RISM studies were utilized to reposition existing FDA-approved marine drugs and to give new molecular insights. This study also provides the best option in the discovery of CoVID-19 pharmaceuticals and rapid drug discovery for safe and efficient CoVID-19 virus-replication inhibitors.

The drugs Eribulin Mesylate, Plitidepsin, and Trabectedin proposed in this study possess similar pharmacophore features of CoVID-19 main-protease inhibitors. They also possess an excellent binding affinity to CoVID-19 main protease and the ability to interact with key catalytic residues. Solvation studies revealed the complexes formed were stable in bulky-water molecules and with existing, promising bioavailability and safety in humans. The hits discovered in this study have great potential to be translated to clinical use and help reduce the replication of the CoVID-19 virus and its complications, specifically for patients with cancer.

## Figures and Tables

**Table 1:** Inhibitory concentration of proven CoVID-19 main-protease inhibitors.

Chemical Name	IC 50 (μM)
Tideglusib	5.81
PX-12	4.67
Simeprevir	4.86
Boceprevir	5.38
Narlaprevir	3.33
MG-132	5.41
Calpain Inhibitor 11 (ALLM)	6.01
Calapain Inhibitor X11	5.41
Calpetin	4.97
Calapin Inhibitor 1	5.07
GC376	7.52
Ebselen	6.17
Disulfiram	5.03
Carmofur	5.74
Shikonin	4.8
MG-115	5.5

## Data Availability

Not applicable.
